# Molecular characterization of hard tick *Haemaphysalis longicornis* from China by sequences of the internal transcribed spacers of ribosomal DNA

**DOI:** 10.1007/s10493-018-0210-x

**Published:** 2018-02-01

**Authors:** Zhong-Bo Li, Guo-Hua Liu, Tian-Yin Cheng

**Affiliations:** 1 Hunan Provincial Key Laboratory of Protein Engineering in Animal Vaccines, College of Veterinary Medicine, Hunan Agricultural University, Changsha, 410128 Hunan Province People’s Republic of China; 2 Hunan Co-Innovation Center of Animal Production Safety, Changsha, 410128 Hunan Province People’s Republic of China

**Keywords:** *Haemaphysalis longicornis*, ITS regions, China, Hedgehog, Goat

## Abstract

In the present study, the entire first and second internal transcribed spacer (ITS-1 and ITS-2) regions of nuclear ribosomal DNA (rDNA) of *Haemaphysalis longicornis* from China were amplified by polymerase chain reaction. The 45 representative amplicons were sequenced, and sequence variation in the ITS was examined. The ITS sequences of *H. longicornis* were 3644 bp in size, including the part of 18S rDNA, 28S rDNA sequences and the complete ITS-1, 5.8S rDNA and ITS-2 sequences. Sequence analysis revealed that the ITS-1, 5.8S rDNA and ITS-2 of this hard tick were 1582, 152, and 1610 bp in size, respectively. The intra-specific sequence variations of ITS-1 and ITS-2 within *H. longicornis* were 0–2 and 0–2.2%; however, the inter-specific sequence differences among members of the genus *Haemaphysalis* were significantly higher, being 35.1–55.2 and 37–52% for ITS-1 and ITS-2, respectively. The molecular approach employed in this study provides the foundation for further studies of the genetic variation of *H. longicornis* from different hosts and geographical origins in China.

## Introduction

Tick are obligate hematophagous ectoparasites, which usually carry a number of pathogenic microorganisms and parasites, causing diseases in animals and humans (Hajdušek et al. 2013). Ticks belong to three families: Ixodidae, Argasidae and Nuttalliellidae (Burger et al. 2012). Among these three families, the Ixodidae is the most important, and it is consisting of 705 species. Recently, many studies have confirmed that the Ixodidae can transmit a variety of pathogens, including bacteria, helminths, protozoa, and viruses (Greay et al. 2016; Silaghi et al. 2016). *Haemaphysalis longicornis* (Acari: Ixodidae) has a wide distribution in different countries and regions around the world, including China, India, Japan, Vietnam and the far east region of Russia. *Haemaphysalis longicornis* possess a variety of hosts, including sheep, goat, cattle, horse, cat and chickens (Wang et al. 2015).

Genetic variation is a very general phenomenon in metazoans, including ticks. The internal transcribed spacers (ITS) of nuclear ribosomal DNA (rDNA) is a useful molecular marker, which can provide valuable information for investigating population genetic structures, systematics and phylogenetics of hard ticks. The ITS-1 sequences of *Ixodes scapularis* had been used to confirm that the *Ixodes scapularis* constitute a single species in USA (McLain et al. 1995). Furthermore, Chitimia et al. (2009) utilized the ITS-1 sequences as a well-defined genetic marker to analyze molecular characterization of hard and soft ticks from Romania. For example, Song had employed ITS-2 sequences to distinguish *I. holocyclus* isolated from different geographic ranges (Song et al. 2011). Although ITS sequences in a number of ticks have been well studied, there is a paucity of information on ITS sequence variation among populations of *H. longicornis* of socio-economic significance.

The objectives of the present study were to characterize *H. longicornis* from wild hedgehogs and goats in three provinces of China by their ITS sequences and to study genetic variation within and between *H. longicornis* and other hard ticks.

## Materials and methods

### Parasites and DNA extraction

All adult ticks of *H. longicornis* (n = 45) were obtained from wild hedgehogs and goats in three provinces (Henan, Shandong and Hunan) of China. The ticks were washed in physiological saline, identified preliminarily to species based on host preference, morphological characters and predilection sites (capitulum, pedipalp, stigma plate) (Deng 1978), fixed in 70% (v/v) ethanol and stored at − 20 °C until use. Total genomic DNA was isolated from this tick using sodium dodecyl sulphate/proteinase K treatment, followed by spin-column purification (Wizard^®^ SV Genomic DNA Purification System, Promega, Madison, Wisconsin, USA).

### PCR amplification and sequencing

The complete ITS regions including primer flanking 18S rDNA, ITS-1, 5.8S rDNA, ITS-2 and 28S rDNA sequences was amplified by polymerase chain reaction (PCR) from individual tick DNA preparations using two primers, namely ITS1F (5′-TCATAAGCTCGCGTTGATT-3′), ITS1R (5′-AGCTGGCTGCGTTCTTCAT-3′), ITS2F (5′-CGAGACTTGGTGTGAATTGCA-3′) and ITS2R (5′-TCCCATACACCA-CATTTCCCG-3′) (Chitimia et al. 2009). The PCR reactions contained ~ 20 ng of genomic DNA and were carried out in 50-μL reaction volumes containing 25 μL 2 × Phusion EmwealdAmp MAX HS PCR Master Mix (TaKaRa, Dalian, China), 2 μL of each primer, 2 μL DNA and 19 μL of ddH_2_O and 2 µL of DNA sample in a thermocycler (Biometra, Göttingen, German). The PCR conditions were as follow: At the beginning of the preheat temperature 94 °C for 5 min, followed by 35 cycles of denaturation at 94 °C for 30 s; annealing at 54 °C for 30 s, and the extension temperature at 72 °C for 2 min, and a final extension at 72 °C for 5 min. These products of by PCR amplification were detected by 1.5% agarose gel electrophoresis to validate amplification efficiency. The PCR products were sent to BGI-Shenzhen (Shenzhen, China) for sequencing from both directions.

### Complete ITS sequences analysis

The ITS-1 and ITS-2 sequences of *H. longicornis* were determined by comparison with those of *H. longicornis* (GenBank accession number JQ737121). Using the computer program Clustal X 1.81 (Thompson et al. 1997) to align all sequences of ITS regions of 45 strains *H. longicornis*. The formula *D* = 1 − (*M/L*) was used to calculate sequence differences of 45 strains *H. longicornis* isolated from China. In the formula, differently capital represent differently means, where M is the number of alignment positions at which the two sequences have a base in common, and *L* is the total number of alignment positions over which the two sequences are compared (Chilton et al. 1995). Furthermore, the Meg align procedure within DNAStar 5.0 (Burland 2000) was used to analyses sequence similarity of different tick species. Moreover, the Maximum likelihood (ML) method was used for phylogenetic re-constructions. ML analyses were performed using PhyML 3.0 (Guindon et al. 2010), and the GTR + I model with its parameter for the concatenated dataset was determined for the ML analysis using JModeltest (Posada 2008) based on the Akaike information criterion (AIC). Bootstrap support (BS) for ML trees was calculated using 100 bootstrap replicates. To study the phylogenetic relationships with other *Haemaphysalis* species. For ITS-1 region of *H. longicornis*, the *H. parva* (FN_296280), *H. punctata* (FN_296264) and *H. flava* (JQ_737122) were considered into the present study; For ITS-2 region of *H. longicornis*, the *H. doenitzi* (JQ_346685), *H. flava* (JQ_737122) were considered into the present study; The *Rhipicephalus haemaphysaloides haemaphysaloides* (JQ_737126) were considered as the outgroup. Phylograms were drawn using the Tree View program version 1.65 (Page 1996).

## Results and discussion

A total of genomic DNA was isolated from 45 adult female *H. longicornis*, which collected from wild hedgehogs and goats in Henan, Hunan and Shandong provinces, China. The ITS-1 and ITS-2 regions were amplified, and subjected to agarose gel electrophoresis. Amplicon of all samples appeared as a single band, approximately 1600 and 1700 bp in length, respectively.

The sequences of ITS-1 and ITS-2 were 1582 and 1610 bp in size, respectively. These sequences have been deposited in GenBank under the accession numbers: For ITS-1, MF490294–MF490308 (from Hunan province), MF490309–MF490323 (from Shandong province) and MF490324–MF490338 (from Henan province); For ITS-2, MF490339–MF490353 (from Hunan province), MF490354–MF490368 (from Shandong province) and MF490369–MF490383 (from Henan province). The intra-specific sequence variation within *H. longicornis* were 0–2% for ITS-1, and 0–2.2% for ITS-2. However, comparing with other member of *Haemaphysalis*, the results shown that the inter-specific sequence differences among members of the genus *Haemaphysalis* were significantly higher, being 35.1–55.2, 37–52% for ITS-1, ITS-2.

Comparative analysis of different geographical isolates of *H. longicornis* from the same province revealed genetic variations for ITS-1, ITS-2. The result shown that sequence variation in ITS-1 was 0–0.16% among samples from Hunan province, 0–0.58% among samples from Henan province and 0–0.59% among samples from Shandong province. Sequence variation in ITS-2 was 0–0.54% for samples from Hunan province, 0–0.22% for samples from Henan province and 0–0.33% for samples from Shandong province. Among three provinces, the highest variation of gene sequences is Shandong province for ITS-1. However, the highest variation of gene sequences is Hunan province for ITS-2.

**Fig. 1:**
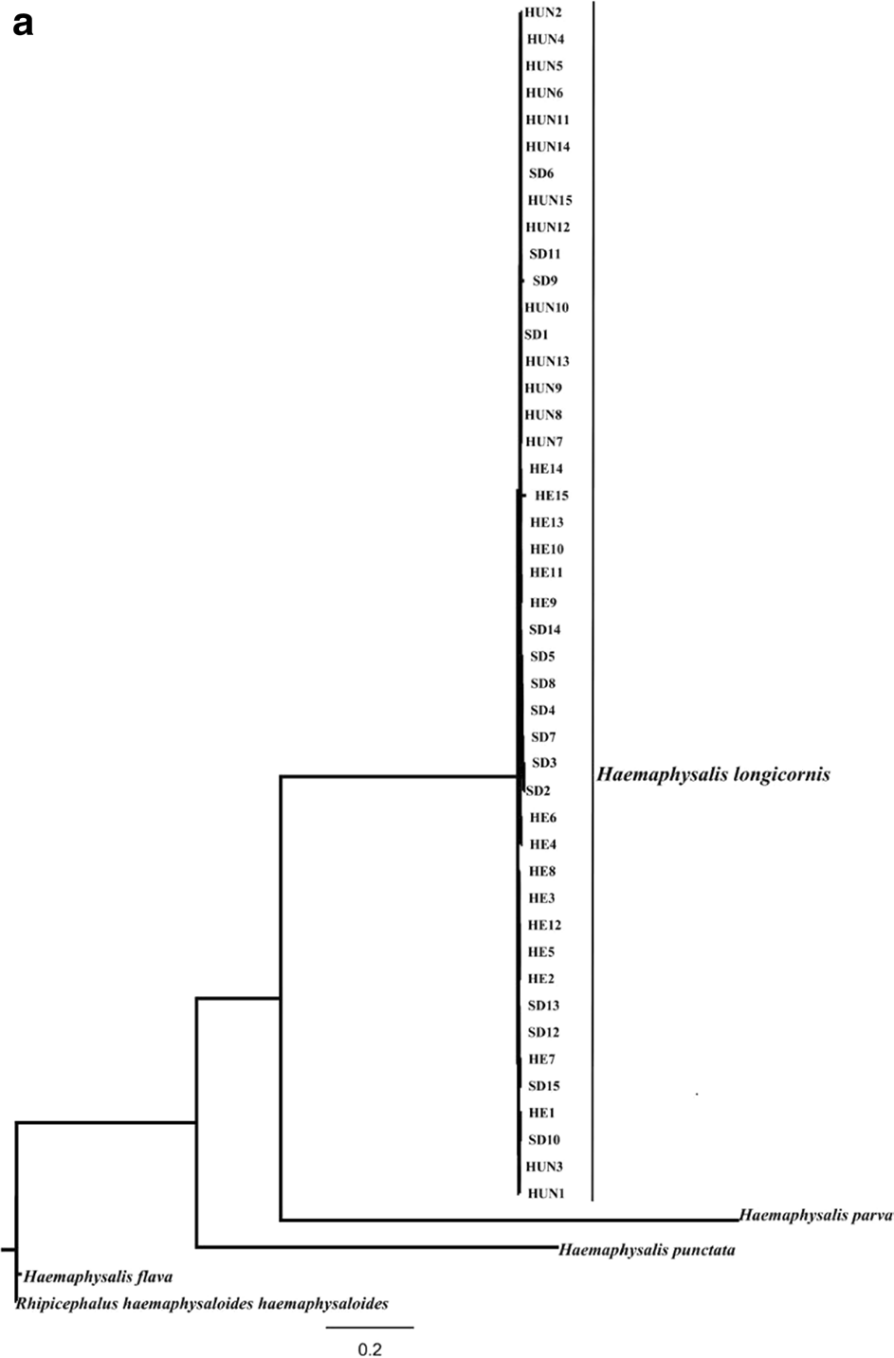

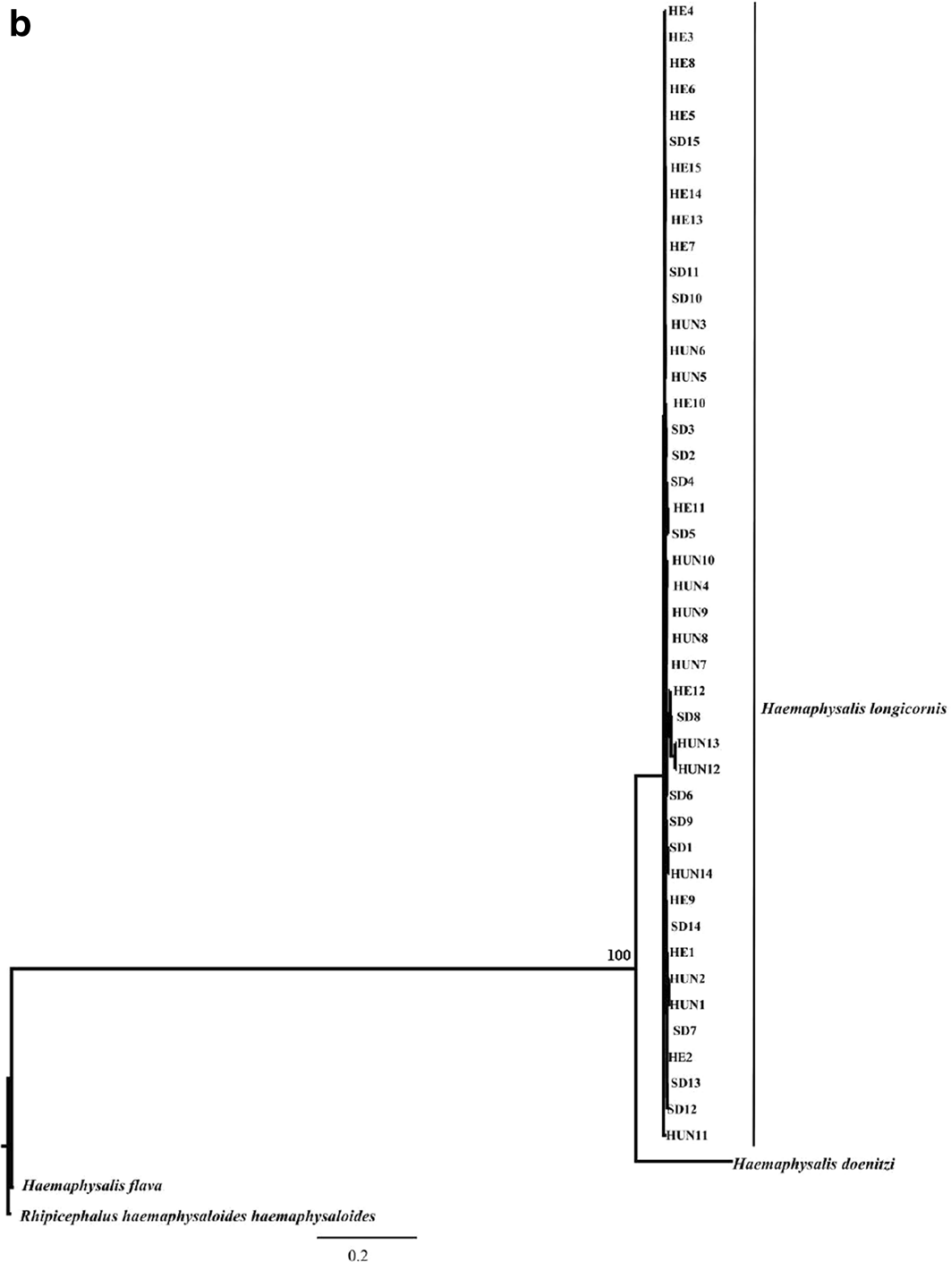
Phylogenetic relationship among *Haemaphysalis longicornis* isolates in China with other Haemaphysalis species inferred by maximum likelihood analyses using the ITS-1 (**a**) and ITS-2 (**b**), with *Rhipicephalus haemaphysaloides haemaphysaloides* as out-group

Many studies have demonstrated that ITS sequences are valuable genetic markers for phylogenetic studies of different groups of ectoparasites, including ticks (such as *Ixodes ricinus*, *Dermacentor marginatus*, *H. punctata*, *H. parva* and *Dermanyssus gallinae*) (Chitimia et al. 2009; Murrell et al. 2001). In the present study, phylogenetic analyses of the sequences of ITS1 and ITS2 among 45 individual *H. longicornis* isolates from China, four other ticks using ML is shown in Fig. 1. In these trees, the *H. longicornis* form monophyletic group with high statistical support (BS = 100), indicated that all present isolates represent hard tick *H. longicornis*.

In conclusion, sequence variations among *H. longicornis* isolates from different geographical localities in China were revealed by sequence analyses of ITS sequences. The molecular approach employed in this study provides the foundation for further studies the genetic variation of *H. longicornis* in different geographical origins in China.
